# A qualitative study to create a framework of Métis understandings of brain health promotion and dementia prevention in Alberta, Canada

**DOI:** 10.3389/frdem.2026.1799514

**Published:** 2026-04-13

**Authors:** Shanaya Fischer, Meagan Ody, Lisa Zaretsky, Jennifer D. Walker, Pamela Roach

**Affiliations:** 1 Department of Family Medicine, Cumming School of Medicine, University of Calgary, Calgary, AB, Canada; 2 Department of Health Research Methods, Evidence and Impact, McMaster University, Hamilton, ON, Canada; 3 Departments of Family Medicine and Community Health Sciences, Cumming School of Medicine, University of Calgary, Calgary, AB, Canada; 4 O’Brien Institute for Public Health, University of Calgary, Calgary, AB, Canada; 5 Hotchkiss Brain Institute, University of Calgary, Calgary, AB, Canada

**Keywords:** brain health promotion, cultural safety, dementia prevention, health equity, Indigenous health, Métis, qualitative research

## Abstract

**Background:**

Indigenous populations experience a higher prevalence of dementia compared to non-Indigenous populations. *Brain Health PRO* is a web-based educational program designed to increase knowledge around dementia risk factors and help create lifestyle changes. The purpose of this study is to understand whether *Brain Health PRO* is relevant to Métis communities in Alberta, Canada.

**Methods:**

Métis participants were recruited across Alberta and interviews were completed using the Métis visiting methodology (Keeoukaywin). Data were co-analysed using Indigenous approaches to reflexive thematic analysis.

**Results:**

Sixty-three interviews with 20 participants were completed between April and November 2024. The analysis generated two main themes which suggest the overall design and delivery of *Brain Health PRO* are not culturally relevant for Métis people in Alberta. The first theme, “Promoting Métis knowledge systems” was demonstrated through discussions of the seven components of *Brain Health PRO* (physical health, cognitive engagement, nutrition, sleep, social and psychological health, vascular health, and vision and hearing), and how these components excluded traditional Métis knowledge. The second theme, “Holistic approaches to risk reduction” described the how Métis knowledge could influence the way each component is presented and understood together in a Métis worldview. The subthemes were: importance of interaction and balance of multiple components; benefits of in-person program delivery; brain health promotion across the lifespan; accessing resources; and the legacy of colonization.

**Discussion:**

Though the seven components of *Brain Health PRO* may be useful for Métis people, the content and delivery of these components lacked Métis knowledge and worldviews. In-person engagement with Métis communities is a crucial next step to co-develop resources that are culturally appropriate.

## Introduction

*The Brain Health Support Program (Brain Health PRO)* is a web-based educational risk reduction program developed for older adults in Canada at risk of cognitive decline. The program is 45 weeks in length, available in French and English, includes lifestyle questionnaires and goal setting, and consists of seven components related to brain health and dementia risk factors. These seven components are: physical activity, cognition, nutrition, sleep, social and psychological health, vascular health, and vision and hearing. Brain Health PRO was created by Canadian brain health and dementia researchers, practitioners and patient-partners. It has not yet been released for public use but there exist plans to implement it in several public health agencies, community organizations and clinical settings across Canada (Canadian Institutes of Health Research, 2026). Although the program was designed to be inclusive, testing of its usability and acceptability has so far been restricted to a small group of highly educated, white older adults (Belleville et al., 2025). Indeed, research is needed to test this program with more diverse populations.

Indigenous Peoples in Canada include the groups that are the first inhabitants of the land including First Nations, Inuit, and Métis people, and their descendants. Each group has distinct histories, federal rights, languages, governance, cultural practices and spiritual beliefs. First Nations are diverse peoples comprising over 600 distinct recognized communities which represent over 50 Nations. Métis people were historically born from post-contact unions between Europeans and First Nations. These unions have formed a distinct language, cultural identity, and unique cultural traditions (e.g., fiddle music, floral beadwork, the Red River Jig) through an ethnogenesis that originated throughout the Red River Valley in central Canada. Inuit are Indigenous people of the north/Arctic regions spanning across four regions: Inuvialuit [Northwest Territories and Yukon, Nunavik (Northern Quebec), Nunatsiavut (Labrador) and Nunavut (Crown-Indigenous Relations and Northern Affairs Canada, 2024)].

Data show the prevalence of dementia is higher in Indigenous versus non-Indigenous populations (Jacklin et al., 2013; Li et al., 2014). Due to lasting effects of colonization and colonial policies, Indigenous people have higher rates of risk factors for dementia such as lower levels of educational attainment, diabetes, and cardiovascular disease (Walker et al., 2020; Henderson et al., 2024). As a result, by 2050, the prevalence of Indigenous people in Canada living with dementia is expected to increase by 273%, compared to non-Indigenous populations which is expected to increase by 187% (Alzheimer Society of Canada, 2024).

In 2015 the Truth and Reconciliation Commission of Canada (TRCC) released 94 Calls to Action urging the federal government, in partnership with all areas of Canadian society and led by Indigenous Peoples, to close these health disparities (Truth and Reconciliation Commission of Canada, 2015). Additionally, Public Health Agency of Canada (2019) calls for additional work to improve brain health and dementia prevention resources for Indigenous people. Repeatedly, community-driven research has identified that culturally safe dementia resources are a priority (Halseth, 2022; Jacklin and Walker, 2020; Eustace et al., 2025; Racine et al., 2022; Lanting et al., 2011; Ody et al., 2022; Webkamigad et al., 2020; Jacklin et al., 2015). Despite these calls to action, little to no culturally specific brain health promotion materials exist for Indigenous people at risk for or living with dementia in Canada (Webkamigad et al., 2020; Ody et al., 2025), and none that are specifically designed for Métis people.

Research on *Brain Health PRO* was recently conducted with the File Hills Qu’Appelle Tribal Council (FHQTC), a service organization representing 11 First Nations in Treaty 4 territory (largely in southern Saskatchewan), to assess the appropriateness of this resource for their communities [File Hills Qu’Appelle Tribal Council (FHQTC), 2024]. From this research, they found that many aspects of *Brain Health PRO* were not culturally appropriate, including the online delivery and the exclusion of traditional knowledge, teachings, and ways of healing. While this report detailed critical insights into brain health promotion materials for File Hills Qu’Appelle communities, it is critical that we further understand these materials in a distinctions-based and culturally specific way (Truth and Reconciliation Commission of Canada, 2015; Public Health Agency of Canada, 2019). A distinctions-based approach requires developing materials that are specific to individual communities or Nations and recognizes that not all Indigenous communities share the same worldview, beliefs and culture. Métis citizens are a frequently underrepresented group in Indigenous health research in Canada (Furgal et al., 2010). Indeed, most studies that aim to identify priorities of dementia care in Indigenous populations only focus on First Nations (Halseth, 2022). As one citizen has shared, *“…I started doing research on it myself. Because all I could find was First Nations-based, a First Nations lens brought into it. How can we provide a Métis lens into care? For that, we need the research”* (Jana Schulz in Alzheimer Society of Canada, 2024, p. 45).

Given the need for culturally safer, distinctions-based brain health promotion materials, the aim of this study was to develop an understanding of how the *Brain Health PRO* could be adapted to a Métis context in Alberta. Specifically, we asked:

1. How are the components of *Brain Health PRO* congruent or not congruent with Métis understandings of healthy brain aging and health promotion?
2. How can *Brain Health PRO* be adapted to be culturally relevant to Métis communities?

## Methods

### Indigenous engagement

The lead author of this work (PR) is a citizen of the Otipemisiwak Métis Government of the Métis Nation within Alberta and has a longstanding partnership on health research with the Métis Nation within Alberta. This project received support from the Métis Nation within Alberta due to the shared recognition of the current barriers and limitations related to culturally safe brain health care for Métis people. A formal letter from the Director of Health at the Métis Nation within Alberta was received at the funding stage of this project as evidence of community support to the funding body.

To understand the conceptualization of culturally safe dementia prevention interventions for Métis people, we worked in tandem with a Métis advisory circle consisting of five individuals with experience working with Métis people living with dementia, who are care partners of Métis people living with dementia, or who are Métis and living with dementia themselves. This advisory circle was established at the outset of the project in February 2024, with many members involved from the point of study design and data collection planning. Members of the advisory circle provided feedback on the interview questions and research methods prior to the commencement of the study, in addition to contributing to the analysis and validation of the results.

When finalizing the results of this study, an infographic and research brief were created to share with the advisory group, participants, and Métis Nation within Alberta for validation and knowledge sharing. As discussed between the Métis Nation within Alberta and the lead author of this work (PR), results of the research are to be first shared with the Nation before being shared with the public (i.e., published in a research journal).

### Recruitment

In partnership with the Métis Nation within Alberta, we purposively recruited participants via their newsletter who had relevant experience. Participants were invited to participate in this study if they met the inclusion criteria: a Métis person living with dementia; a family member/care partner of a Métis person living with dementia; a Métis community member who had experience with dementia; and/or a health researcher with experience in dementia and brain health with Métis people. Additionally, participants had to reside in Alberta. Participants emailed the research team expressing interest, and the research team confirmed eligibility before scheduling the first interview. All participants provided informed written or explicit oral consent before the start of the first interview and were provided with a cash honoraria for each individual interview. This study was approved by the University of Calgary Conjoined Health Research Ethics Board (REB23-1015).

### Data collection

Participants were offered the choice of participating in focus groups or individual interviews either virtually (via video conferencing) or in person. Interviews were conducted using Keeoukaywin, ‘the visiting way*’*, a methodology rooted in Métis ways of being, with relationality centered in each interaction (Gaudet, 2019). In this methodology, the researcher situates themselves as a visitor, and reflects how their own identity, history, kinship, and culture influence the research and our responsibility to each other. Researchers used semi-structured interview guides developed by the research team and the advisory group. Interviews were split into three separate sessions. Interview one included an overview of *Brain Health PRO* and conversations to understand Métis perspectives on health, aging, and dementia. Interview two explored the seven components of *Brain Health PRO* to understand them from a Métis perspective and discuss cultural relevance. Specifically, we asked about the following: physical health, cognitive engagement, nutrition, sleep, social and psychological health, vascular health, and vision and hearing. During interview three, participants watched a module from the *Brain Health PRO* website. We asked participants to share their thoughts on the overall *Brain Health PRO* program design and delivery. The module, Social and Psychological Health, was chosen by the research team since it demonstrated multiple aspects central to *Brain Health PRO*, such as brain development education, an interactive activity, and tips on how to improve brain health.

### Analysis

All interviews were audio recorded using digital audio recorders external to the video conferencing software and transcribed verbatim using a secure professional transcribing service approved by the University of Calgary ethics review boards (Rev.com). Transcripts were anonymized and verified by the research team. All names and identifying details were removed to protect the anonymity of the participants. Transcripts were managed using NVivo 14 (Lumivero, 2023) for management throughout analysis. We incorporated an Indigenous approach to thematic analysis (Clarke et al., 2019; Drawson et al., 2017) which prioritizes reflexivity, resiliency, relationality and responsibility. An Indigenous approach to analysis prioritizes collaboration, Indigenous knowledge, and a strengths-based approach that ensures the findings have value to the communities (Drawson et al., 2017).

A reflexive thematic analysis was used to generate codes and themes (Braun and Clarke, 2006). One research assistant (SF) immersed themselves in the data through repeated listening to the audio recordings and readings of the transcripts. They then completed initial coding and analysis of the data by identifying descriptive codes. Two more members of the team (MO and LZ) completed a second review of the descriptive coding, ensuring all transcripts were double-coded. Analysis then continued by thematically synthesizing the descriptive codes into larger themes and discussed with the Principal Investigator (PR). Disagreements over analyses and emergent themes were resolved through peer debriefing discussions with the research team and advisory group. The generated themes were presented to the participants and the advisory committee for member checking, and to ensure rigorous data collection, analysis and establish trustworthiness of the data (Lincoln and Guba, 1985).

### Positionality

The team is made up of Indigenous and non-Indigenous researchers committed to enhancing Indigenous health outcomes through ethical research practice. The Principal Investigator and senior author (PR) is a Métis Scholar and citizen of the Métis Nation within Alberta. She holds a Canada Research Chair in Indigenous health systems safety, has extensive qualitative methods expertise, and has been working with families living with dementia for 20 years. The first author (SF) is a Cree scholar descending from Cowessess First Nations. She holds a Master’s in Social Justice and Human Rights where she focused on systemic barriers like income inequality and Indigenous rights. As the primary interviewer and the first coder in this study, her Indigenous heritage and interest in systemic barriers likely influenced her relationships and interactions with participants and shaped her initial coding scheme. The second author (MO) is a non-Indigenous researcher and holds a Master’s in Community Health Sciences. The third author (LZ) is a non-Indigenous researcher with experience working with Métis communities to help advance understandings of dementia and brain health. She holds a Master’s in Community Health Sciences. The fourth author (JW) is a Haudenosaunee member of the Six Nations of the Grand River. She holds a Canada Research Chair in Indigenous Health Data and Aging and leads a community-driven Indigenous research program at McMaster University focusing on aging and dementia research. The positionality of the study team brings together Indigenous and non-Indigenous understandings of health, wellness, and research that inform the analysis and interpretation of the results.

## Findings

Participant demographics are summarized in Table 1. Twenty Métis people in Alberta participated in this study. Of these, 18 participants completed three separate interviews, while two participants joined one focus group followed by two individual interviews each as a result of scheduling difficulties. All participants opted to participate virtually. Interviews occurred between July 4, 2024 and October 22, 2024, and the one focus group took place in April 2024. Sixteen participants were family members of someone living with dementia, and four were professionals working in dementia care (e.g., nurses). The average age of participants was 47 years old (range: 25 years to 70 years), and most participants were women (*n* = 17). Participant names have been replaced with pseudonyms for anonymity.

**Table 1:** Demographics.

Participant	Gender	Age	Indigenous identity	Experience with dementia
DARLENE	Woman	38	Métis	Grandmother had dementia
SELENA	Woman	34	Métis	Grandmother and aunt had dementia
SIMON	Man	38	Métis and Cree	Grandfather and uncle had dementia
JOSEPHINE	Woman	36	Métis	Nurse working in dementia care
CYNTHIA	Woman	39	Métis	Caregiver for grandfather with dementia, aunt had dementia
SAMSON	Man	53	Métis	Dementia runs in maternal family—grandmother, mother, aunts, uncles, cousins
SIENNA	Woman	58	Métis	Nurse working in dementia care and two family members with dementia
MELISSA	Woman	30	Métis	Professional working in long-term dementia care
VIOLET	Woman	66	Métis and Cree	Partner had dementia
WILLOW	Woman	65	Métis and Cree	Father had dementia
SARAH	Woman	70	Métis and Cree	Mother had dementia, aunt has dementia
CELESTE	Woman	25	Métis	Grandfather had dementia
HAVEN	Woman	55	Métis	Grandma had dementia, mother experiencing signs of dementia
AUDREY	Woman	65	Métis	Mother has dementia
PAIGE	Woman	46	Métis	Mother has dementia
JANE	Woman	39	Métis	Nurse working in dementia care
CARLENA	Woman	56	Métis	Two aunts with dementia
NAOMI	Woman	36	Métis	Grandmother had dementia
CLIFF	Man	51	Métis	Dad and uncle have dementia, grandmother had dementia
HAZEL	Woman	48	Métis	Grandmother and great grandmother had dementia

A total of 57 audio recordings were used for analysis between October 2024 and February 2025. The interviews ranged from 14 min to 119 min with an average length of 41 min. Through the completion of the analysis, two overarching and interconnected themes were generated from the data: (1) Promoting Métis knowledge systems, and (2) Holistic approaches to risk reduction. The themes built on a coding scheme that began with descriptive codes about health, aging, and Métis culture. Themes, subthemes, and example codes are summarized in Table 2. These themes are described in more detail below and quotes have been edited to enhance clarity and readability.

**Table 2:** Themes, subthemes, and codes.

Themes	Subthemes	Codes
Promoting Métis knowledge systems	Physical	Cultural activities
	Cognitive engagement	Cultural activities Community
	Nutrition	Cultural activities Community
	Sleep	Trauma Spiritual health
	Social and psychological health	Relationship to land Intergenerational caregiving Spiritual health
	Vascular health	Health promotion
	Vision & hearing	Spiritual health Respect of elders
Holistic approaches to risk reduction	Importance of interaction and balance of multiple components	Aging process Holistic Health promotion
	Benefits of in-person program delivery	Holistic Feedback
	Brain health promotion across the lifespan	Intergenerational Preserving knowledge
	Accessing resources	Barriers Income
	The legacy of colonization	Cultural safety Trauma Residential school Racism

### Promoting Métis knowledge systems

Participants noted that western ways of understanding brain health promotion in content and delivery excluded traditional Métis knowledge. Most *Brain Health PRO* components had no mention of traditional Métis ways of living in their examples, images, or suggestions.

#### Physical health

When participants were asked what physical health means to them, they spoke about many cultural activities that supported their physical wellbeing such as berry picking, hunting, and jigging (a traditional form of dance).

*“There’s like berry picking. Like, you can walk and pick berries and like you’re raising your arms. You’re doing that hunting. It’s physical activity too.”* (Darlene, Interview 2).

Not only were these traditional ways of physical activity excluded in the content, but many participants noted the lack of resources (e.g., financial, geographical) to take part in western physical activities (e.g., gym, fitness classes). Further excluded from *Brain Health PRO’s* discussion on physical health was the emphasis Métis people placed on being surrounded by community, such as berry picking or jigging together, rather than focusing on individual fitness activities.

#### Cognitive engagement

When participants were asked what cognitive engagement meant to them, they spoke of quilting, beading, music and fiddle playing, oral storytelling, learning and speaking the Michif language, and spirituality. The most common response to cognitive engagement from a Métis perspective was the importance of sharing and passing on traditional knowledge and oral storytelling. Similarly, many participants highlighted the significance of having connection to their culture to promote cognitive wellness.

*“Well, I think story telling, just learning different stuff. So if you’re out learning about plants you have to learn about when to pick it, how to pick it, how to use it, how to not to use it, when to store it. Like it’s just so intensive that it makes you have to be alert all the time if you want to learn it. And I think story telling, teaching others, you know, how to do stuff. Just interaction, you know, even at community gatherings, like interacting with other people.”* (Paige, Interview 2).

These examples of cognitive engagement conflict with those provided in Brain Health PRO, which instead includes activities like computer skills, cards, and piano. Furthermore, this topic explicitly states that having a discussion with another person is less cognitively engaging, and thus beneficial, than other activities such as digital photography. This section also has a large focus on memory strategies, and a whole chapter on “writing things down” which excludes Métis traditional ways of knowing that focus on oral storytelling. Although Brain Health PRO includes a chapter on “Music and Culture” in the Cognitive Engagement section, it discusses activities like art exhibits, theater shows, music classes, and book clubs, which are not congruent with Métis cultural activities.

#### Nutrition

Participants were explicit in stating that the Canada Food Guide, highlighted in *Brain Health PRO* as the guide to follow for healthy brain aging, does not align with Métis culture. Participants shared the importance of traditional diets and living off locally sourced foods. Many described hunting, harvesting, and eating together as a community. Berries, moose meat, and bannock were foods that came up frequently amongst participants. Furthermore, many participants spoke about being intolerant to many of the foods suggested by the Canada Food Guide such as dairy and starches, and concerns about diabetes.

*“If our culture, our people went back to their traditional foods, we wouldn’t have all the diabetes, we wouldn’t have all the chronic illnesses. Because our diet, our traditional diet is a healthy diet and it doesn’t follow the food guide. And then they had lots of fruits, vegetables, meats. A lot of the wild meat was made into a big soup pot and you ate it when you’re hungry, right? It was always on the stove and you ate it when you needed to.”* (Sienna, Interview 2).

#### Sleep

Participants generally tended to agree with *Brain Health PRO*’s content regarding sleep and how it influences healthy aging and brain health. Some participants, however, shared that dreams have cultural and spiritual significance for them.

*“Oddly enough, I do get the dream that does come true kinda like a déjà vu. My mom used to dream, and it would- it would happen. So yeah, lots of déjà vu. And that’s somebody telling me I’m on the right path.”* (Carlena, Interview 2).

Others spoke about how intergenerational trauma can cause significant sleep disruptions and how an educational intervention like *Brain Health PRO* may need to address this in order to be more relevant for Métis people.

*“I’ve seen a lot of trauma and, and then I dreamt, uh, a lot of trauma.”* (Willow, Interview 2).

#### Social and psychological health

Participants mentioned how social health is much broader in a Métis context. Not only is community and intergenerational relationships central to everything, but social health also includes relationships to the land, animals, and Mother Earth. Many mentioned the importance of Elders. For example, aging is not necessarily associated with a loss of strength, but rather a gaining of knowledge. It is seen as something to be admired and respected. Elders are carriers of knowledge and spiritual guides who bring meaningful contributions to the community. As such, losing Elders to dementia and the loss of this intergenerational knowledge is dire. One participant mentions how this is particularly impactful for Métis:

*“You know, we only came into being when Canada was colonized, you know, we are a young history. So we have more to lose by losing our elders.”* (Hazel, Interview 1).

One of the largest and most apparent gaps in *Brain Health PRO*’s representation of health was the exclusion of spiritual health. Nearly all participants mentioned that spiritual health is a critical pillar of health and wellness and noted its exclusion in *Brain Health PRO*. Spiritual health for participants was varied and included praying to Creator, smudging, and participating in cultural ceremonies such as pipe ceremonies. It is also important to recognize that through the ongoing process of colonization many Métis people were converted to Catholicism and therefore the incorporation of spiritual health needs to incorporate the diversity of Métis spiritual beliefs.

*“So spiritual wellness, it could be different for everyone, you know? Including Métis people who come from a, a range of backgrounds. But for me, it’s a connection to the processes of the land and a connection to each other and just a knowledge of like the cycle of things. The ongoing cycle of things and the impermanence of things. And just trying to keep things in balance.”* (Melissa, Interview 1).

*“Spiritual health involves, again, your whole being. So it’s your spirit, obviously. Um, and to feed your spirit, to have a healthy spirit, I feel it’s necessary to have daily communion with a creator, which I do.”* (Sarah, Interview 1).

#### Vascular health

Overall, participants agreed with the content pertaining to vascular health in *Brain Health PRO*. Participants spoke about the importance of having a healthy heart and eating well and being physically active to support this.

*“Uh, vascular health is exactly what Brain Health PRO says. (laughs) I mean, it’s, uh, taking care of your, your heart and your circulatory system, your organs, everything internal that is making you function and live your life and take care of your body.”* (Jane, Interview 2).

However, some participants were confused by the word “vascular,” indicating the need for appropriate terms using accessible language and incorporating Métis ways of knowing.

*“When I hear the word vascular, it doesn’t mean anything to me. But when you put it in perspective then I’m hoping that I have a healthy heart, healthy lungs.”* (Carlena, Interview 2).

#### Vision and hearing

Some differences shared between Métis experiences with vision and hearing versus that demonstrated in *Brain Health PRO* were that visual experiences, visions, and hearing voices can be understood as spiritual and culturally significant. Some participants also expressed that changes in vision and hearing can be understood to be more spiritual as opposed to a deficit or mental illness. This indicates the need for knowledge translation materials that are culturally relevant and acknowledge that some risk factors may be both culturally significant and a risk factor at the same time.

*“I think maybe that can maybe make up for a loss of one, the other, or some loss, because they’re a little more spiritual. And I don’t mean in a religious sense, I mean like a connection with the earth and the animal kingdom. So I guess I’m looking at it that way, because they have that kinda connection with the spiritual world. I think if an individual in that community was to lose some or all of one of your senses, that maybe that spiritual connection kinda maybe... Not makes up for it, but kinda compensates, maybe.”* (Cliff, Interview 2).

### Holistic approaches to risk reduction

Participants described the possibility for Métis knowledge to influence how each component is presented and how they could be understood together in a Métis worldview.

#### Importance of interaction and balance of multiple components

Many described how a single cultural activity could encompass multiple aspects of health. For example, berry picking or medicine picking not only includes connecting to culture, but also connecting to land, being physically active and gathering nutritious food, and social engagement. One participant spoke of how she went medicine picking with her granddaughter, which added another component to the activity: intergenerational connection and teaching.

*“I went medicine picking yesterday with my granddaughter. I consider that physical activity, gardening, chores with the animals. We had to clean out the chicken coop and pick eggs. And so all of those things. And to me that, again, like I think of it in a holistic measure, you know, I’m increasing food security and I am teaching the little ones about skills that will be good for the rest of their lives.”* – (Haven, Interview 2).

Some participants mentioned the teachings of the medicine wheel (a symbol of balance, interconnectedness, stages of life, and/or aspects of health; Dababneh et al., 2025), and how health and healthy aging is understood as encompassing all four quadrants of the medicine wheel. As one participant described, central to this understanding of health was ensuring all components of health are in balance. Indeed, many participants used the word “balance” when talking about health, and the different components of health.

*“I know I went to a workshop where they did a medicine wheel workshop and you were able to sort of grasp where you were with ideas of what mental and emotional health looked like and then you were able to sort of see how you were out of balance with the wheel. And I use that on a regular basis to say, “Okay, I’m not feeling great today. Is part of my wheel out of balance,” and nine times out of ten it is.”* (Haven, Interview 1).

Also important to Métis understandings of healthy aging and wellness is how different components of health influence and interact with each other. Two participants in particular spoke of how emotional and physical health underly overall wellness.

*“So I find with aging and of a different culture, or belief system, which everybody really has, the health system is just too busy to bother. So, good health to me is to have emotional health throughout. That will give you everything else. If you don’t have emotional health and acceptance from those in the community that are helping you, in whatever community you end up in, whatever environment. And respect for who you are, that’s how you will age well.”* (Violet, Interview 1).

*“Um, physical health, well, it’s because we are whole, whole creatures, but physical health is just one part of our whole, whole being, which effects everything. So, if our physical health is not good, it effects every other part of us, too.”* (Sarah, Interview 2).

#### Benefits of in-person program delivery

Further emerging from this theme were concerns and suggestions relating to the delivery of the program. Participants noted the delivery of the program was highly individual in nature, which is not congruent with the importance of community in Métis culture. Many participants suggested having in-person group events instead of, or in addition to, the online format. Some participants also noted how this would fulfill the dual purpose of social connection while also educating about dementia prevention, thus taking a more holistic approach to dementia risk reduction.

*“They’re encouraging, you know, getting out there and having a social network. But you can’t get that sitting in front of this computer.”* (Cynthia, Interview 3).

*“I think you’d want to do both online and in person. […] It would be, let’s meet, let’s talk about everything. Let’s talk about this process that’s available to you. Are you interested in something like that? Um, because for me, it’s important to gain as much knowledge as you can and share it with others.”* (Audrey, Interview 3).

#### Brain health promotion across the lifespan

Participants described that tailoring brain health promotion materials to younger adults and youth would align with Métis understandings of health, which emphasizes wellbeing across the life-span and more upstream preventative approaches.

*“So, if we can get the younger ones, in their 20s and younger even, to buy into this program and take care of themselves and each other, then we can maybe stop this from happening at all, right?”* (Samson, Interview 3).

#### Accessing resources

Throughout the interviews, participants noted difficulty in accessing resources when trying to improve their health and brain health and thus identified a need to address this underlying issue if we are to address brain health in Métis communities. For example, transportation, availability, and cost were all noted barriers when participants spoke of accessing social and psychological supports, physical activities, nutritional food, and vision and hearing resources.

*“For example, last night’s supper was pulled pork, corn and peach pie. (laughs). So that’s definitely not part of the food guide, but we got everything we needed in there, and it satisfied our hunger. It was what we could afford and it was what we liked.”* (Haven, Interview 2).

*“…How does a senior afford a CPAP machine for $2,800? Where does that come from? Seniors’ benefits do not cover that. So there is a financial piece again, everything from housing, food, prior costs, everything went back to financial.* (Violet, Interview 1).

A nurse who worked in dementia care also spoke of their surprise when they entered the field at how many resources a patient needs, and how important it is for them to be able to access all those services.

*“But I don’t know, it’s, it’s like a, it’s a big thing. Like, you need physio, you need occupational therapy. You need speech therapy. You need swallow assessments. And you need home care, you need accessibility. You need help and support in the community. You need to make sure that resources are available to you. Like, do you need transportation? […] Like, it takes a whole whack of team, even pharmacists and doctors, to make this care plan specific for individuals living with dementia to ensure that their quality of life is maintained throughout.”* (Jane, Interview 3).

Participants also spoke of how the cost of living was a barrier to engaging in traditional ways of living and caring for aging family members.

*“… I am always planning for when our parents are older, they can live here and we’ll move into the basement and that kinda thing. I know other families, like Indigenous families I work with, it’s kinda like, “Well, we can look after Mom for a little bit, but we cannot have her forever. “You know, like they have little kids and were working. And I think, like, cost of living is through the roof.”* (Josephine, Interview 1).

#### The legacy of colonization

Participants shared concerns and experiences of racism and discrimination when accessing health services, especially those who present as more visibly Indigenous. For example, one participant shared their experience watching their grandma access healthcare.

*“…Because my grandma was quite dark, so just like experienced racism, different things like that. Just not having the best experiences within the healthcare system and then at the end of her life, she’s dependent on people that she does not trust for help.”* (Naomi, Interview 1).

Relatedly, many participants mentioned the impact of colonization on the health of their nation. When one participant was asked what a Métis perspective on dementia would be, they shared that it was a “western view,” and that this is due to colonization, and being disconnected from the heritage.

*“So not a lot of culture was passed on that way, and it wasn’t for the last like three or four decades that we’ve been trying to dig it back out. And that’s from colonization, right, like just the perspective and it just being, like, hush-hush or, ‘No, you don’t have rights because you married out.”* (Josephine, Interview 1).

## Discussion

This study explored Métis perspectives on a brain health promotion program. Similar to findings from First Nations communities [File Hills Qu’Appelle Tribal Council (FHQTC), 2024], the components of *Brain Health PRO* are not culturally relevant to, nor congruent with, Métis culture and people in Alberta, Canada. Furthermore, a holistic approach to healthy aging is missing in *Brain Health PRO*’s content, design, and delivery and in how it fails to acknowledge barriers to accessing different supports and services.

The impact of culture on health outcomes is supported by previous research done in an Alberta First Nations context. In working with Cree and Blackfoot community members, Oster et al. (2014) identified that connection to culture is inseparable from health. The current study also aligns with previous research that suggests a holistic approach to health is imperative for Métis people and other Indigenous communities [Ody et al., 2022; Webkamigad et al., 2020; File Hills Qu’Appelle Tribal Council (FHQTC), 2024].

One way in which Métis culture was lacking in *Brain Health PRO* is in the exclusion of traditional ways of engaging in physical activity such as berry picking and living off the land. Given that physical inactivity is one of the largest risk factors for dementia (Macdonald et al., 2015; Nguyen et al., 2024), it is imperative that brain health promotion materials include culturally relevant physical activities. Indeed, previous research suggests that increasing physical activity among Métis people would be more successful if it also involved cultural activities (Ryan et al., 2018).

Participants noted that important cultural cognitive engagement activities including the importance of sharing traditional histories and oral storytelling, along with reconnecting to culture and the traditional Michif language were also excluded. Oral storytelling and sharing circles are culturally appropriate styles of education (Webkamigad et al., 2020). Therefore, it would be apt to ensure brain health promotion materials serve the dual purpose of cognitive engagement and education through culturally appropriate methods such as these.

Métis culture and understanding of nutrition is significantly different from the Canada Food Guide, and the importance of eating locally grown berries, meat, and bannock is lacking in *Brain Health PRO*. The use of the Canada Food Guide in *Brain Health PRO* may be further inappropriate to use with Indigenous communities given the historical residential school-based experiments used to create this guide (e.g., Tennant, 2021). Participants’ view of nutrition included the role of food sharing and eating together as a community. Eating together also serves the dual purpose of social engagement; an important risk factor covered in *Brain Health PRO*. Future brain health promotion materials should include Indigenous food and knowledge systems which center around traditional foods and cultural values that would be framed in a more holistic way when thinking about prevention and risk reduction.

The importance of traditional intergenerational relationships, community, cultural ceremony, connection to culture and spirituality are ignored in components of social and psychological health. Previous research has also shown how intergenerational relationships are an important factor of healthy aging for First Nations adults (Cornect-Benoit et al., 2020). This includes participating in culture and traditional teachings, once again pointing to the importance of including culturally relevant examples in brain health promotion materials. This also relates to study participants’ suggestions of creating materials that are relevant across the lifespan, which further ties into Métis views of holistic health and wellness.

A notable gap in *Brain Health PRO* was any content about spiritual health. Most participants explicitly named spiritual health as one of the main pillars of brain health and wellness. This re-emphasizes findings from previous research on dementia care with Indigenous communities, which has also suggested the importance of spirituality [Pace, 2020; Forbes et al., 2013; File Hills Qu’Appelle Tribal Council (FHQTC), 2024; Jacklin et al., 2015]. Spirituality can also mitigate several dementia risk factors. For example, research has shown that high rates of physical activity are associated with a high level of self-reported spirituality in Métis adults (Ryan et al., 2018). The incorporation of spiritual health into dementia prevention interventions may therefore have an impact to other dementia risk factors. Other culturally relevant teachings missing from *Brain Health PRO* is the relevance of visions or hearing voices as potentially being a part of the spiritual significance of Métis culture. Furthermore, the lack of sharing by participants in relation to the vision and hearing module of *Brain Health PRO* may also be because vision and hearing loss may not be as significant risk factors for dementia for Indigenous populations as they are for non-Indigenous populations (Walker et al., 2020). Future brain health promotion materials for Métis communities may consider the amount of emphasis placed on vision and hearing, not only from the perspective of Métis spirituality but also to make sure that materials are specific to the risk factors for the population they are meant to serve. More in-depth examination of specific relevance of risk factors for individual populations will be required for future resource development. These types of materials will need to be co-designed with communities so that the information about the risk factors in these cultural and spiritual contexts can be accessible and relevant.

Although the section on vascular health resonated with participants, for some it only resonated when the term “vascular” was described in more relatable language by the researcher such as using the term “heart health.” Again, this speaks to the importance of using relevant and appropriate language in brain health promotion materials, not only in terms of using plain language but also in terms of using culturally appropriate terms (Webkamigad et al., 2020).

As described, participants noted the lack of Métis culture in *Brain Health PRO*’s delivery. This includes the exclusion of Métis people in its imagery. Research has demonstrated that use of familiar, relevant and elaborate visuals can be an effective education style for Métis and other Indigenous people (Lanting et al., 2011). Furthermore, many participants disliked the online, individualistic, lengthy format of *Brain Health PRO*, and suggested smaller, in-person, Indigenous-led gatherings. Indeed, dementia communication is likely to be more effective when done in shorter durations in more culturally appropriate ways, such as storytelling or sharing circles (Lanting et al., 2011; Webkamigad et al., 2020). This misalignment of language and of imagery supports the critical nature of appropriate knowledge translation and knowledge sharing tools in order for future educational interventions to have the desired impact.

Many participants described how colonization has impacted their ability to engage in cultural activities, and therefore dementia risk reduction activities. The Truth and Reconciliation Commission of Canada (TRCC) was established to acknowledge the impact of the historical and ongoing colonization of Indigenous peoples. In 2015, the TRC released 94 Calls to Action to address these impacts (Truth and Reconciliation Commission of Canada, 2015). Calls to Action 18 to 24 address gaps in health outcomes experienced by Indigenous communities. Colonization has indeed impacted Indigenous communities across Canada by changing and restricting access to traditional food systems (Malli et al., 2023; Henderson et al., 2024). This is thought to be a key factor in the higher rates of diabetes, a known risk factor for dementia (Biessels et al., 2006), in Indigenous populations when compared to non-Indigenous populations in Canada (Cunningham, 2009). Previous research has demonstrated that Indigenous language knowledge was significantly associated with lower diabetes prevalence (Oster et al., 2014). Using Indigenous language rates as a proxy for culture, we can extrapolate that increasing the cultural relevance, or cultural congruency, of healthcare and health promotion services may be a powerful preventative approach to advancing Indigenous health outcomes.

The legacy of colonization has left many Indigenous people, including the Métis participants in this study, experiencing anti-Indigenous racism in many facets of life, including when accessing health services (Roach et al., 2023). This may lead to healthcare avoidance and poor health outcomes (Cooke and Shields, 2024) and addressing these gaps in health outcomes is an urgent healthcare priority (Truth and Reconciliation Commission of Canada, 2015). The importance of Indigenizing health systems and services, including creating culturally safe dementia materials cannot be understated.

### Strengths and limitations

There are several limitations to this work. The first stems from the virtual nature of the interviews and focus groups. While participants were offered the choice to meet in person, almost all decided to meet online. This is likely due to the variety of locations participants live, which are often great distances from each other and major cities. Virtual interviews and focus groups have been shown to be an acceptable method of visiting with Métis citizens in Ontario (Simms et al., 2025). However, this may come at a cost to Indigenous approaches to interviewing. Keeoukaywin typically involves face-to-face interaction, and sharing of food and culture (Gaudet, 2019). However, Keeoukaywin still guided our interview methodology in terms of repeated visits, slowing down to build relationships, and ensuring reflexivity and accountability throughout. The use of online platforms with Métis communities in Alberta deserves further investigation since many participants expressed that a strictly online platform is not a preferred method of delivery for a brain health prevention intervention.

Because of the use of an online platform for interviews, participants were only given the opportunity to look at a small portion of *Brain Health PRO* content via screen sharing over video conference and were only shown one video module out of several. This did not give them the opportunity to adequately experience the entirety of the program and provide feedback on the program as a whole. Another limitation of this study was that none of the participants were identified as living with dementia. Though they all had experience with family members with dementia or working professionally with people living with dementia, it is important that future research makes a concerted effort to include the voices of those living with dementia. Finally, Métis people in Alberta are a diverse group with different cultural experiences, connections, and places of residence (e.g., living in Métis settlements versus urban centers), these results may not apply broadly to all Métis people. Future work should aim to recruit participants from diverse locations and settings to further explore Métis concepts of dementia risk reduction and prevention. There are also several strengths to this work that enhanced the rigor of the study. These strengths include the positionality and lived experience of the study team. The deep relationships with community provide the opportunity to create trusted spaces where participants can share openly. The repeated interviews and data collection meant there was the opportunity to ensure thick description of the data, triangulate the data and analysis with the research team, the participants, and the advisory group therefore enhancing trustworthiness and dependability. Ongoing relationships with the community also means that work can be carried forward in a reciprocal and relational way to meet community priorities.

## Conclusion

The findings of this study demonstrate the importance of co-designing and co-creating brain health promotion materials with community members. Through interviews with Métis citizens in Alberta, it was shown that the seven components of *Brain Health PRO* are not culturally congruent with Métis understandings of health and brain health. Furthermore, the design and delivery of this platform did not resonate with important aspects of Métis culture such as intergenerational teachings, community relationships, or holistic wellbeing. Future work in developing brain health promotion materials must include Métis voices from the very beginning phases of development in order to ensure cultural congruency.

## Data Availability

The datasets presented in this article are not publicly available due to the nature of the qualitative data collected, ethics approval, and agreements with community partners. Requests to access the datasets should be directed to the corresponding author at pamela.roach@ucalgary.ca.
